# LncRNA UCA1 acts as a sponge of miR-204 to up-regulate CXCR4 expression and promote prostate cancer progression

**DOI:** 10.1042/BSR20181465

**Published:** 2019-05-02

**Authors:** Chang He, Xuwei Lu, Fan Yang, Liang Qin, Zhuifeng Guo, Yang Sun, Jiawen Wu

**Affiliations:** Department of Urology, Shanghai Minhang Hospital, Fudan University, Shanghai 200199, People’s Republic of China

**Keywords:** CXCR4, miR-204, prostate cancer, UCA1

## Abstract

Prostate cancer (PCa) is a devastating malignant disease with a poor prognosis. The aim of current study is to investigate the role of lncRNA-urothelial carcinoma associated 1 (UCA1) in the progression of PCa. We evaluated the expression levels of UCA1 in a total of 16 benign prostatic hyperplasia tissues (BPH) and 40 PCa tissues, as well as PCa cells. The functional regulatory effects of UCA1 were investigated using a series of cell function approaches. Our data showed that UCA1 is frequently overexpressed in PCa tissues compared with BPH tissues (*P*<0.01). Moreover, the higher expression of UCA1 was observed in patients with Gleason score ≥8 (*P*<0.05). In consistent, we found the expression levels of UCA1 was higher in the PCa cell lines PC-3, LnCaP, and DU-145 than in the normal prostate epithelial cell line RWPE-1 (*P*<0.01). Functionally, we found knockdown of UCA1 in PC-3 significantly suppressed cell growth and invasion of PC-3, while overexpression of UCA1 in DU-145 cells promote cell growth and invasion. Mechanistically, UCA1 overexpression permitted activation of CXCR4 oncogenes through inhibition of miR-204 activity, as evidenced by the positive association of these two genes with UCA1 levels and inverse correlation with miR-204 expression in PCa tissues. Luciferase activity assay further confirmed the targetting relationship between UCA1 and miR-204, CXCR4, and miR-204. The up-regulation of UCA1 in PC-3 cells significantly impaired the inhibitory effect of miR-204 on CXCR4 expression. Taken together, our research revealed that UCA1 works as an oncogene by targetting miR-204. The UCA1-miR-204-CXCR4 regulatory network regulated the growth and metastasis of PCa, providing new insight in the management of patients with such malignancy.

## Introduction

Prostate cancer (PCa) is the second most common malignancy and a major leading cause of cancer death amongst men worldwide [1]. Although the 5-year survival rates of PCa steadily increase in the United States, the mortality of PCa has been increasing every year globally, particularly in East Asia [2, 3]. While there is considerable morbidity and mortality as a result of PCa, a significant population of men with PCa have indolent and slow growing disease that does not require intervention. Distinguishing between patients who would benefit from active surveillance, those who require definitive primary treatment, and those who require treatment escalation is a challenging and evolving issue. Therefore, the discovery of new molecular targets for PCa may help to understand the pathogenesis, prognosis, or treatment of PCa.

LncRNAs, a class of noncoding RNAs with the length ranging from 200 nts to almost 100 kilobases, play vital roles in cancer development [4]. Moreover, accumulating evidences showed that dysregulation of lncRNA expression attributes a tumor-suppressor or an oncogenic role to lncRNAs affecting the clinicopathological appearance, prognosis, and outcome of in the PCa [5, 6]. The lncRNA, urothelial carcinoma-associated 1 (UCA1) has been identified as an oncogene that enhances cell proliferation, inhibits apoptosis and promotes cell cycle progression in several types of cancer [7]. For instance, UCA1 was shown to activate Wnt/β-catenin signaling pathway to promote progression and epithelial-mesenchymal transition (EMT) in oral and breast cancer [8, 9]. In gastric cancer, UCA1 has been shown as an early detection serum maker, and the induction of UCA1 by TGF-β leads to the enhanced invasion and migration in gastric cancer cells [10, 11]. However, the role of UCA1 in PCa remains largely unknown.

CXCR4 had gained tremendous attention over past decades since it was overexpressed in a multiple cancer types and contributes to their malignant behaviors [12–15]. A recent meta-analysis has shown that higher positive rate of CXCR4 was associated with T3/4 stage, the presence of lymph node or bone metastasis of PCa, and poor survival of patients with PCa [16]. In line with clinical findings, *in vitro* studies showed high levels of CXCR4 induce a more aggressive phenotype in PCa cells [17, 18]. Interestingly, the bone environment, in which CXCR4′ligand SDF1α is particularly highly expressed, is also the most common metastatic site of PCa. Moreover, metastatic PCa cells localized in the bone metastatic lesions express higher SDF1α/CXCR4 levels relative to the cells present in primary tumors and lymph node metastatic lesions [19–23], suggesting that the activation of the SDF1α/CXCR4 pathway may play a pivotal role in PCa bone metastases. In the present study, we found that UCA1 is overexpressed in PCa cancer tissues, as well as PCa cells. In consistent, knockdown or overexpression of UCA1 is able to inhibit or promote the proliferation and invasion of PCa cells. Mechanismly, we found that UCA1 functions as miR-204 sponge to up-regulate CXCR4 expression. Our study for the first time to show that UCA1-miR204-CXCR4 regulatory network plays is a key role in the development of PCa, highlighting this pathway may serve as a potential therapeutic target in PCa patients.

## Materials and methods

### Clinical tissue samples

All tissues were collected at the Department of Urology, Shanghai Minhang Hospital between January 2015 and December 2017. Patients have received a detailed pathological assessment. All patients have accepted consent for the use of all samples. The present study was also approved by the Medical Ethics and Human Clinical Trial Committee of the Shanghai Minhang Hospital.

### Cell culture and transfection

All cell lines, including PC-3, DU-145, LNCaP, and RWPE-1, were purchased from the American Type Culture Collection. According to the manufacturer’s instructions, the cells were cultured in the RPMI1640 medium with 10% FBS in 37°C with 5% CO_2_.

### Vectors and transfection

LncRNA UCA1 siRNA, CXCR4 siRNA, and miR-204 mimics were purchased from GenePharma (Shanghai, China). UCA1 was amplified from the cDNA of PC3 cells using PrimerSTAR (TaKaRa) and cloned into the pcDNA3.1(+) vector. All cells were transfected with 100 nM miR-204 mimics, UCA1 siRNA, CXCR4 siRNA, or 2 µg pcDNA3.1(+)-UCA1 expression vector using Lipofectamine 2000 reagent (Invitrogen) according to the manufacturer’s instructions. The WT and MT 3′UTR of CXCR4 or the UCA1 fragment containing the miR-204 binding sites were synthesized and then cloned into the luciferase reporter vector p-Luc.

### Cell viability assay

Cell viability was determined by CCK-8 assay. Different kinds of cells were seeded in 96-well plate with 5000 cells/well. After 1, 2, 3, and 4 days, cells were treated with CCK-8 reagent for 1 h in the incubator. Then optical density was detected by microplate reader at 450 nm in triplicate, and the mean value of absorbance was referred to the quantity of viable cells.

### Transwell cell migration/invasion matrigel assay

Transwell assay was performed to measure cell migration and invasion ability. Put Matrigel Matrix aliquot on ice at 4°C to thaw. Mix Matrigel Matrix (final concentration of 1 mg/ml) with RPMI-1640 medium. Gently swirling to mix the solution and place the tube on ice. Then add 100 μl of diluted Matrigel Matrix to Transwell insert. Incubate the 24-well plates with the coated Transwell inserts at 37°C for at least 1 h. Carefully remove the remaining liquid from the Transwell insert. Cells were suspended in serum-free DMEM medium containing 0.1% bovine serum albumin. Total 500 µl complete medium was added to the 24-well plate. Then, 5 × 10^4^ cells were seeded in Transwell chambers and incubated for 24 h. Cells on the upper surface of the filter were completely removed. Cells on the lower surfaces of the membrane were washed two-times with PBS and fixed with 95% ethanol for 10 min, then stained with 0.1% crystal blue solution for 10 min and taken pictures under a microscope.

### RNA immunoprecipitation assay

RNA-IP was performed using a kit from Active Motif (Carlsbad, CA, U.S.A.) following the manufacturer’s protocol. PC-3 and DU-145 cells were collected and lysed in RIPA lysis buffer. The total cell protein extract was then incubated with RIP wash buffer containing magnetic beads conjugated with human anti-Ago2 antibody (Millipore) or mouse immunoglobulin G (IgG) control. Then the samples were digested with proteinase K, and RNA was extracted from the beads using TRIzol. Then performed qRT-PCR analysis to measure the presence of the miR-204 and UCA1. The primers are available in the Table 1.

**Table 1:** Primer sequence

Name	Sequence
UCA1-F	CCACACCCAAAACAAAAAATCT
UCA1-R	TCCCAAGCCCTCTAACAACAATGAC
miR-204-F	TCGTGGACTTCCCTTTGCATTTGATG
miR-204-R	GATGGTGCAAT
U6-F	ATTGGAACGATACAGAGAAGATT
U6-R	GGAACGCTTCACGAATTT
CXCR4-F	ACATTGGGATCAGCATCGACTC
CXCR4-R	GGCTCCAAGGAAAGCATAGAGG
E-cad-F	TGCCCAGAAAATGAAAAAGG
E-cad-R	GTGTATGTGGCAATGCGTTC
N-cad-F	ACAGTGGCCACCTACAAAGG
N-cad-R	CCGAGATGGGGTTGATAATG
Vimentin-F	CAACCTACAGGAAGCTTCTGGA
Vimentin-R	AGTCTTCAATTCTCTGGGTAGT
MMP9-F	AAGGATGGGAAGTACTGGCGAT
MMP9-R	GCGCCCAGAGAAGAAGAAAAGC
GAPDH-F	GACTCATGACCACAGTCCATGC
GAPDH-R	AGAGGCAGGGATGATGTTCTG

### RNA isolation and quantitative real-time PCR

Total RNA was isolated using TRIzol reagent (Invitrogen). cDNA was synthesized using the PrimeScript™ RT Master Mix Kit (TaKaRa, RR037A) or TIANGEN^®^miRcute Kit (KR211). Quantitative RT-PCR was measured using SYBR^®^ Premix Ex Taq™ (TaKaRa, RR420A) or TIANGEN^®^miRcute Plus miRNA qPCR Kit (FP411). The relative expression of UCA1 and miR-204 was calculated by the 2^−ΔΔ*C*^_T_ method.

### Western blot analysis

For WB, all cells were collected and lysed in RIPA lysis buffer. Proteins were resolved on SDS-PAGE gels and transferrd proteins to negative controls (NC) membranes and then followed by standard WB protocols. The primary antibodies used were: CXCR4 (Abcam, ab124824), β-actin (Abcam, ab8226). β-Catenin (Abcam, ab32575), ERK1/2 (Abcam, ab184699), pERK1/2 (Abcam, ab223500), pAKT (Abcam, ab38449), and AKT1/2/3 (Abcam, ab179463).

### Luciference reporter assay

HEK-293T cells were cultured in 96-well plates and cotransfected with 50 nM miR-204 mimic (or NC), 50 ng of luciferase reporter vector (containing UCA1-WT, UCA1-MT, CXCR4 3′UTR WT, or CXCR4 3′-UTR MT), and 5 ng of renilla luciferase plasmidusing Lipofectamine 2000 (Invitron). After 48 h, the luciferase activity was determined by using Dual Luciferase Assay System (Promega, Madison, WI, U.S.A.) following to the manufacturer’s information.

### Statistical analysis

All results are performed as the mean ± S.E.M. Student’s *t* test, the Mann–Whitney U test, the Kruskal–Wallis test and the χ^2^ test were used to measure the differences amongst different groups. The Kaplan–Meier method and log-rank test were conducted to determine differences in survival rates. A Cox proportional hazard analysis was used to evaluate the prognostic factors in univariate and multivariate analyses. A *P* value <0.05 was considered statistically significant.

## Results

### UCA1 is up-regulated in PCa tissues and cell lines

**Figure 1:**
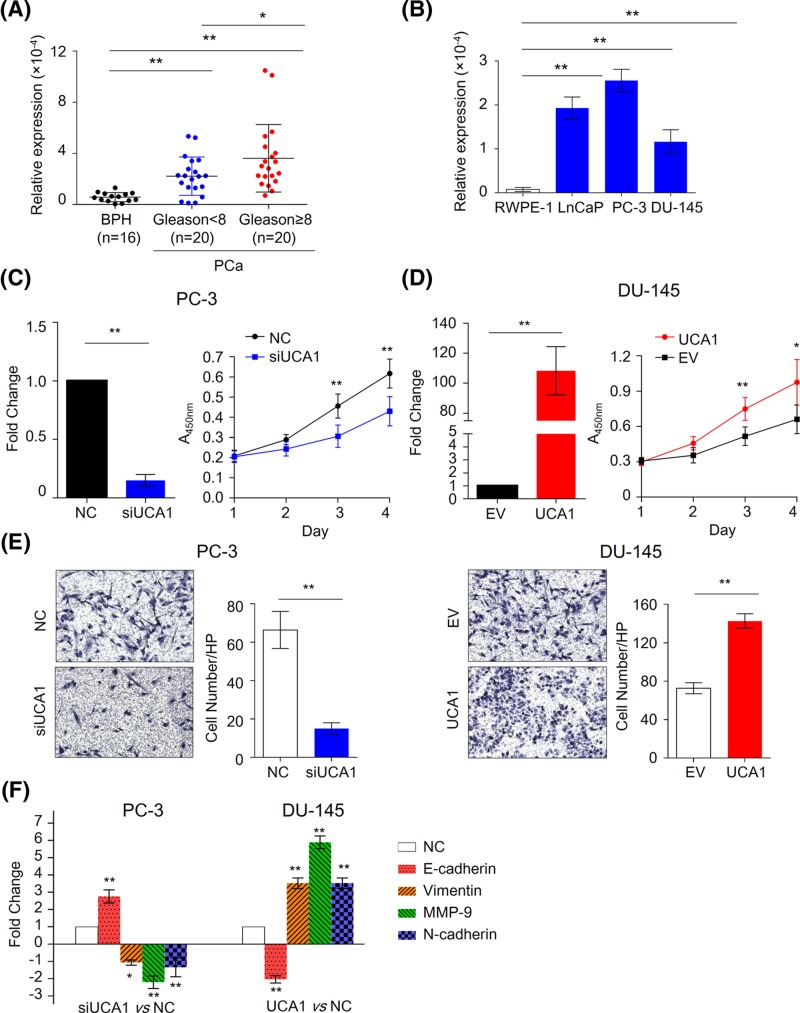
UCA1 plays an oncogenic role in PCa (**A**) The expression level of UCA1 in BPH and PCa tissues. *P<0.05, **P<0.01, Mann–Whitney U test was used to PCa and BPH tissues. (**B**) The expression level of UCA1 in normal prostate epithelial cell line RWPE-1 and PCa cell lines LnCaP, PC-3, and DU-145 cells. **P<0.01, Mann–Whitney U test was used to compare PCa cells and normal cells. The treated or control cells were harvested 24 h after transfection with siRNA or plasmids. (**C**) The relative expression level of UCA1 in PC-3 after treatment of siUCA1 (left figure, *n*=3). The growth curve for UCA1 knockdown PC-3 cells and control cells were evaluated by CCK-8 assay (right figure, *n*=6). **P<0.01, Mann–Whitney U test was used to compare control (NC) and UCA1 knockdown cells. (**D**) The relative expression level of UCA1 in DU-145 after overexpression of UCA1 (left figure, *n*=3). The growth curve for UCA1 overexpression cells and control cells were evaluated by CCK-8 assay (right figure, *n*=6). **P<0.01, Mann–Whitney U test was used to compare control (EV: empty vector) and UCA1 overexpression cells. (**E**) The transwell-matigeal invasion assays were performed to evaluate the effect of down-regulation (upper figure) or up-regulation (bottom figure) of UCA1 on the capacity of invasion in PCa cells. **P<0.01,Mann-Whitney U test was used to compare control and treated cells. EV: empty vector. (**F**) qPCR was performed to evaluated the several metastasis-associated genes expression in the UCA1 knockdown PC-3 cells or UCA1 expressing DU-145 cells. * P<0.05,**P<0.01,Mann-Whitney U test was used to compare control and treated cells. EV, empty vector.

To explore the biological function of UCA1 in PCa, we initially measured relative expression of UCA1 in PCa and benign prostatic hyperplasia (BPH) tissues. The results clearly showed that the expression level of UCA1 was higher in PCa tissues than in BPH tissues (*P*<0.01, Figure 1A). Moreover, the higher expression of UCA1 was observed in patients with Gleason score ≥8 (*P*<0.05), suggesting that the up-regulation of UCA1 was associated with disease progression.

We subsequently examined UCA1 expression in three human PCa cell lines and in the normal prostate epithelial cell line RWPE-1. In consistent, we found the expression levels of UCA1 was higher in the PCa cell lines than in the normal prostate epithelial cell line (*P*<0.01, Figure 1B). These findings indicated that UCA1 is up-regulated in PCa tissues and cell lines and may play an important role in the development of PCa.

### UCA1 promotes proliferation and invasion of PCa cells

To investigate the biological functions of UCA1 in PCa cells, we performed cell viability and transwell-invasion assay. Since PC-3 cells have highest level of UCA1, while DU-145 have lower level of UCA1, we constructed UCA1 siRNA and overexpression plasmids to manipulate UCA1 expression in PC-3 cells and DU-145 cells. As shown in Figure 1C, knockdown of UCA1 significantly suppressed cell growth of PC-3 cells (*P*<0.05). In contrast, overexpression of UCA1 in DU-145 remarkably promote cell growth (*P*<0.05, Figure 1D). Similarly, we found the capacity of cell invasion was impaired in UCA1 knockdown PC-3 cells but enhanced in UCA1 overexpression DU-145 cells (*P*<0.01, Figure 1E). To confirm the effect of UCA1 on cell invasion, we also measured several metastases associated markers in UCA1 knockdown or overexpression PCa cells by using qPCR (Figure 1F). In line with invasion assay, we found E-cadherine is up-regulated, while Vimentin, MMP-9 and N-cadherin is down-regulated in UCA1 knockdown PC-3 cells (*P*<0.05). In contrast, the overexpression of UCA1 in DU-145 showed increased level of E-cadherin but decreased level of Vimentin, MMP-9 and N-cadherin (*P*<0.01), validating the aggressive role of UCA1 in cancer metastases.

### UCA1 regulates CXCR4 expression in PCa cells

**Figure 2:**
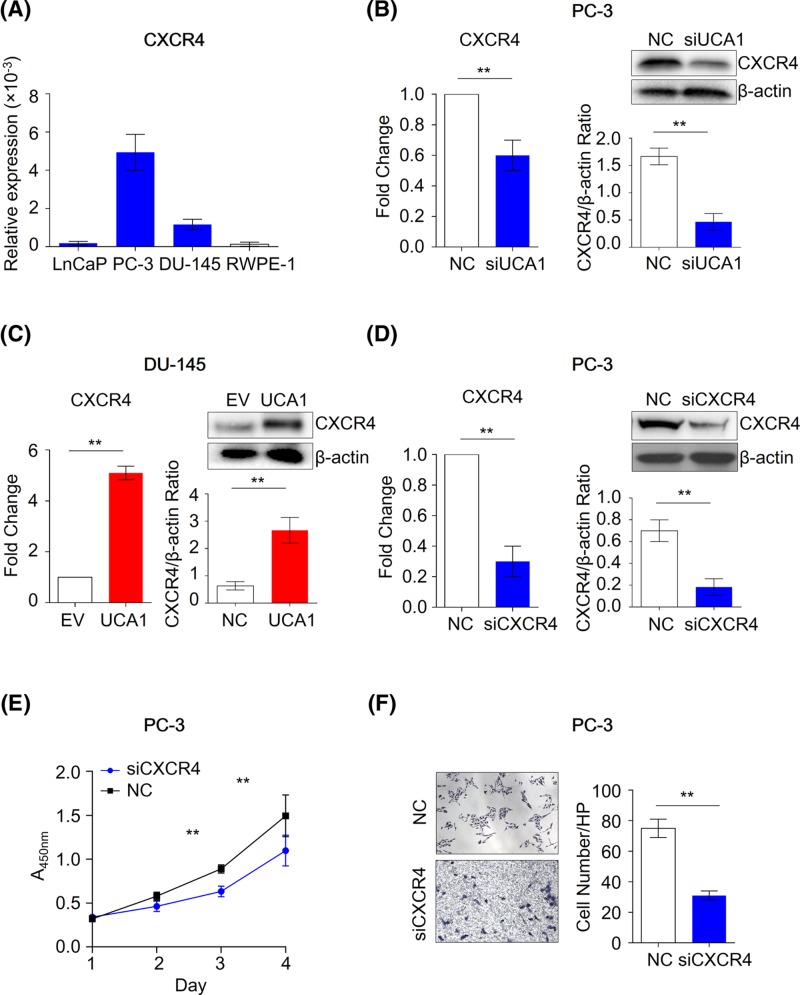
UCA1 regulates CXCR4 expression in PCa cells (**A**) The expression level of CXCR4 in normal prostate epithelial cell line RWPE-1 and PCa cell lines LnCaP, PC-3 and DU-145 cells (n = 3). **P<0.01,Mann-Whitney U test was used to compare PCa cells and normal cells. (**B**) The mRNA (left figure) and protein (right figure) level of CXCR4 in NC and UCA1 knockdown PC-3 cells (n = 3). **P<0.01,Mann-Whitney U test was used to compare NC cells and UCA1 knockdown cells. (**C**) The mRNA (left figure) and protein (right figure) level of CXCR4 in the control (NC) and UCA1 overexpression DU-145 cells (n = 3). **P<0.01, Mann–Whitney U test was used to compare NC cells and UCA1 overexpression cells. (**D**) The mRNA (left figure) and protein (right figure) level of CXCR4 in the control (NC) and CXCR4 knockdown PC-3 cells (*n*=3). **P<0.01, Mann–Whitney U test was used to compare NC cells and CXCR4 knockdown cells. (**E**) The CCK-8 assay (*n*=6) and (**F**) transwell-matrigeal assay (*n*=3) was performed to evaluate the biological effect of CXCR4 knockdown in PC-3 cells. **P<0.01, Mann–Whitney U test was used to compare NC cells and CXCR4 knockdown cells.

Since UCA1 was overexpressed in PCa tissues and cells lines and associated with malignant biologic phenotype, we next interrogate its mechanisms for the oncogenic role of UCA1 in PCa. Recent study showed that UCA1 is able to regulate CXCR4 in osteosarcoma cells [12]. However, the exact mechanisms for the interaction between UCA1 and CXCR4 remain unclear. Since CXCR4 plays a central role in the development of PCa, particular in PCa bone metastasis, we therefore investigated the regulatory effect of UCA1 on CXCR4 expression. Interestingly, similar to UCA1, the expression of CXCR4 was higher in PC-3 cells, but lower in DU-145 cells (Figure 2A). The knockdown of UCA1 leads to the inhibition of CXCR4 expression in PC-3 cells (Figure 2B), while overexpression of UCA1 results in the significant up-regulation of CXCR4 in DU-145 cells (Figure 2C), suggesting that UCA1 is able to regulate CXCR4 in PCa.

To further understand the biological impact of UCA1 on CXCR4, we successfully inhibited CXCR4 by siRNA interference in PC-3 (Figure 2D). We confirmed that knockdown of CXCR4 in PC-3 leads to the suppressed cell viability and invasion (Figure 2E,F), suggesting that UCA1 may exert its oncogenic function, at least in part, through CXCR4.

### UCA1 functions as a competing endogenous RNA of CXCR4

**Figure 3:**
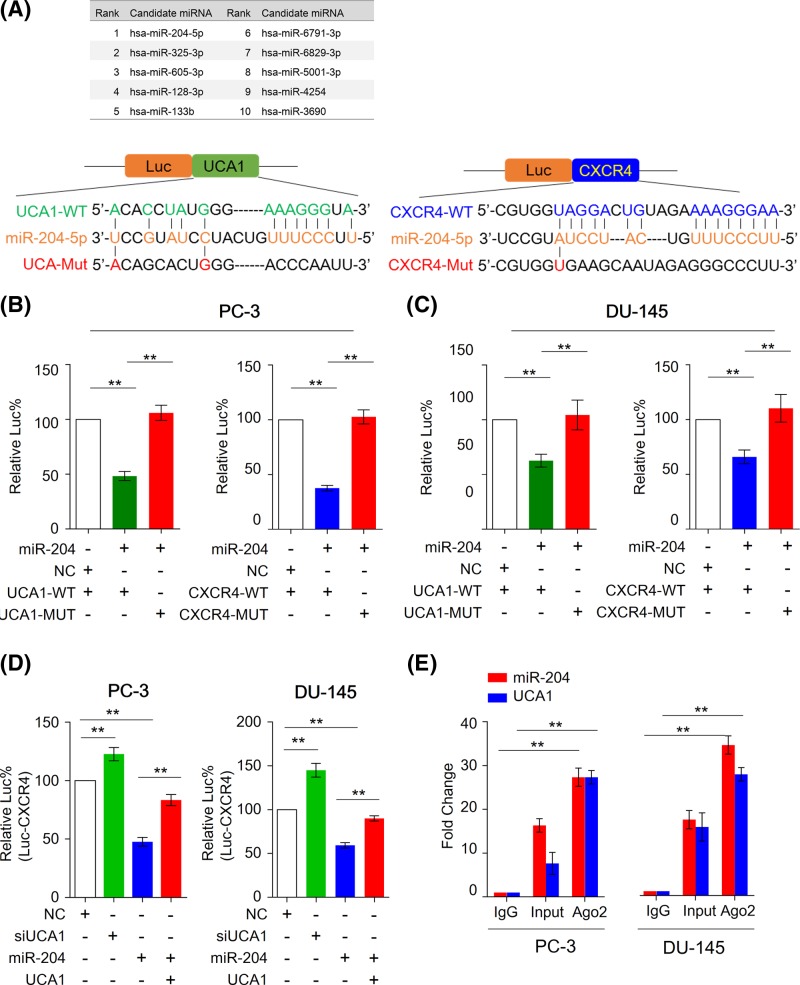
miR-204 binds to both UCA1 and CXCR4 *in vitro* and *in vivo* (**A**) Schematic representation of luciferase reporter constructs. The PGK promoter drives constitutive transcription of a chimeric mRNA containing the firefly luciferase coding sequence fused to the wild-type or mutated UCA1 and CXCR4 3′-UTRs. (**B**) and (**C**) Relative activity of the luciferase gene fused with the wild-type or mutant UCA1 3′-UTR in PC-3 and DU-145 cells. (**D**) Relative activity of the luciferase gene fused with wild-type of CXCR4 in PC-3 and DU-145 cells. The data was normalized to renilla luciferase activity. The data are represented as means ± S.D. from separate transfections (*n*=3). Statistical analysis was performed using Mann–Whitney U test to compare fold changes between transfection and control groups. All statistical tests were two-sided. **P<0.01. (**E**) RNA binding protein immunoprecipitation (RIP) assay was performed to investigate the interaction between UCA1, miR-204, and Ago2 protein. The pull-downed RNA was used to analyzed relative expression of miR-204 and UCA1 in PC-3 and DU-145 cells (*n*=3). **P<0.01. Mann–Whitney U test was used to compare fold changes between control and Ago2 groups. Luc%, percentage of relative luminescence; Mut, mutant type; WT, wild type.

Based on previous reports that UCA1 has the capacity to regulate RNA molecules by sponging several miRNAs such as miR-193a[13], miR-195[14], miR-143[15], miR-204[16], miR-182[17], we presumed that UCA1 might regulate CXCR4 expression by inhibiting miRNA function. By utilizing microRNA.org algorithm (www.microRNA.org), we searched several UCA1 targetted miRNAs in the 3′-UTR region of CXCR4 and identified miR-204as candidate with highest mirSVR score amongst predicted targets (Figure 3A), suggesting that UCA1 may promote CXCR4 expression through inhibiting miR-204.

To confirm our hypothesis, we first validated that miR-204 can functionally bind to 3′-UTR region of UCA1 in PCa cells. As shown in Figure 3B and C, we cloned miR-204 binding region of UCA1 into luciferase report plasmid to establish wild type of 3′-UTR report plasmids. In addition, we also mutated miR-204 binding sites and established mutant 3′-UTR regions of UCA1. PC-3 and DU-145 cells were transiently transfected with these constructs along with miR-204 mimics or negative controls (NC). In line with our previous results, miR-204 mimics treatment significantly suppressed luciferase activity of the reporter genes containing wild-type 3′-UTR regions of UCA1, but no inhibitory effects were observed in mutated sequence, supporting that miR-204 can bind to 3′-UTR of UCA1.

Furthermore, we investigated that whether miR-204 can bind to 3′-UTR regions of CXCR4, and whether such interaction will be inhibited by UCA1 in PCa. Similar to UCA1, we cloned miR-204 binding region of CXCR4 into luciferase report plasmid. In line with our assumption, the treatment of miR-204 mimics inhibited luciferase activity of the reporter genes that containing 3′-UTR regions of CXCR4 in PC-3 and DU-145 cells. Moreover, coexpressed UCA1 and miR-204 has no inhibitory effects on luciferase activity in PCa cells, indicating that UCA1 may sponge miR-204 to inhibit its inhibitory effects on CXCR4 (Figure 3D). In human, Ago2 (Argonaute-2) has been revealed as the only member of Argonaute family that can binds to miRNAs and their complementary RNA targets, and has catalytic activity to regulate small RNAs guided gene silencing processes, highlighting its essential role in RISC complex [24]. Therefore, to further confirm the physical interaction between UCA and miR-204, we performed RNA immunoprecipitation (RIP) assay. As shown in Figure 3E, both miR-204 and UCA1 are able to bind to Ago2 protein in PCa cells.

### UCA1 up-regulates CXCR4 expression via suppression of miR-204 activity

**Figure 4:**
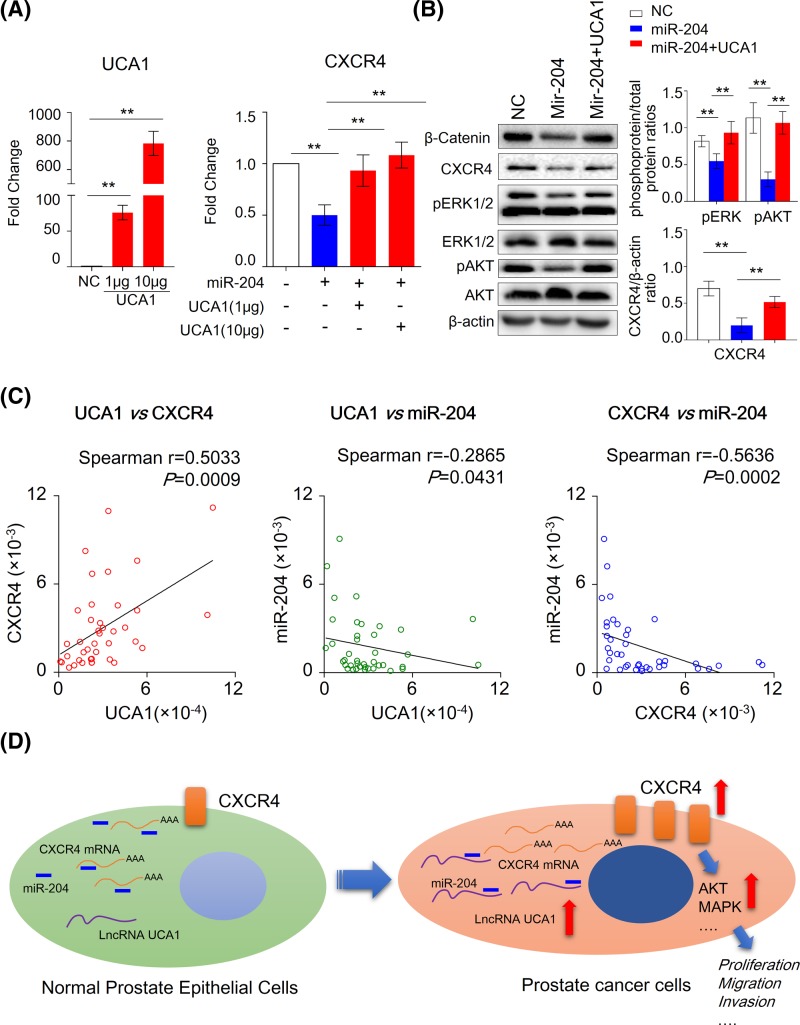
The up-regulation of UCA1 impairs the inhibitory effect of miR-204 on CXCR4 expression (**A**) The left panel showed the expression level of UCA1 in the PC-3 treated with 1 μg, 10 μg of UCA1 plasmids or control plasmids. The right panel showed mRNA level of CXCR4 in control and PC-3 cells treated with miR-204 alone or together with UCA1 plasmids (1 or 10 μg). (**B**) The protein level of β-catenin, pERK1/2, pAKT in control and PC-3 cells treated with miR-204 alone or together with 1 μg of UCA1 plasmids. Mann–Whitney U test was used to compare relative ratio between control and treated groups. (**C**) The expression correlation of UCA, CXCR4, and miR-204 in PCa tissues (*n*=40). Spearman’s rank correlation (r) was used for the correlation analysis. (**D**) A proposed mechanistic model as to how UCA1 functions as a miR-204 sponge and regulates CXCR4 via inhibiting miR-204 activity. The persistent up-regulation of UCA1 sequestered miR-204 and thereby lead to the accumulation of CXCR4 signaling to drive colorectal tumor growth.

To test our hypothesis, we measured CXCR4 expression in PC-3 cells transfected with miR-204 alone or together with CXCR4. In line with our assumption, treatment with miR-204 mimics led to decreased mRNA and protein expression levels of CXCR4, while UCA1 overexpression attenuated this inhibitory effect of miR-204 on CXCR4 expression (Figure 4A). Furthermore, we have found that treatment with miR-204 mimics could not only significantly inhibit CXCR4 expression but as well suppressed its downstream signaling pathway, including Wnt, MAPK, and AKT in PC-3 cells (Figure 4B). However, such inhibitory effect of miR-204 was impaired by UCA1 overexpression.

To further validate our *in vitro* results that UCA1 regulated CXCR4 through miR-204 sponge activity, we investigated the expression correlation between miR-204, UCA1, and CXCR4 in PCa tissues. We noticed that UCA1 expression was negatively correlated with miR-204 expression in PCa (r = −0.2865, *P*=0.0431), suggesting that the loss of function of miR-204 may be not only due to its lowered expression but also due to intimate association with up-regulated UCA1 expression in this malignancy (Figure 4C). As expected, we also found miR-204 expression negatively correlated with CXCR4 (r = −0.5636, *P*=0.0002) in cancer tissues, indicating that miR-204 could suppress CXCR4 expression *in vivo*. Notably, we observed UCA1 overexpression was significantly associated with up-regulation of CXCR4 in PCa (r = 0.5033, *P*=0.0009), highlighting the clinical significance of these results, as these suggest that the up-regulation of UCA1 abrogates the tumor suppressive effect of miR-204 on its downstream target CXCR4 in PCa.

## Discussion

PCa is one of the most common causes of cancer death in the male population in the world. Despite of significant improvement in outcomes of patients with early stage by surgical prostatectomy, radiotherapy, hormone therapy, or immunotherapy, the treatments for the advanced patients are still challenging. High rates of metastasis and cancer-associated mortality are major cause of poor prognosis in PCa patients. Hence, there is an urgent need to find novel mechanisms involved in PCa, which may provide new perspectives on management of PCa patients.

In the present study, we, for the first time, describe UCA1-miR204-CXCR4 regulatory network as a novel pathway that contributes to development of PCa (Figure 4C). We have made several novel observations. First, we discovered that UCA1 is frequently up-regulated in PCa, and this significantly correlated with disease progression. Second, from a biological perspective, we demonstrated that UCA1 and promoted aggressiveness of PCa cell lines. Third, we unraveled a novel mechanism that UCA1 acts as a competing endogenous RNA (ceRNA) of CXCR4 by competing for miR-204. Considering the crucial role of CXCR4 in development of PCa, our results for the first time revealed the therapeutic importance of UCA1 in this malignancy.

Recent work showed lncRNA began to emerge as natural miRNA decoys. Although we have witnessed the discovery of numerous lncRNAs in the past two decades, only a small portion of them have been identified functionally, and very few of them have been validated to function as ceRNAs in cancers [25]. In PCa, the up-regulation of lncRNA GAS5 was reported to inactivate the AKT/mTOR signaling pathway through targetting miR-103. HOTAIR can target HER2 mRNA by binding miR-331-3p and then modulates the depression of HER2 to promote the development of PCa [26]. In addition, lncRNA PVT1 was shown to promote EMT via suppressing miR-186 to activate Twist1 in PCa. UCA1 has been extensively reported as a miRNA sponge in various cancer. For example, Wu et al., showed that UCA1 promotes lung cancer cell proliferation and migration through miRNA-193a/HMGB1 axis [27]. Moreover, UCA1 promotes mitochondrial function of bladder cancer via the miR-195/ARL2 signaling pathway [28]. In addition, UCA1 can interact with miR-182 to modulate glioma proliferation and migration by targetting iASPP [29]. In the present study, we used online prediction program to find candidate miRNAs, which reported previously as UCA1 target miRNA, in the 3′-UTR region of CXCR4. We identified miR-204 as potential miRNAs that links UCA1 and CXCR4. MiR-204 was demonstrated as tumor suppressor in various kind of cancer, and its functions involves cell growth, apoptosis, migration, and invasion [30, 31]. Therefore, UCA1 may function, in part, through inhibition of miR-204 in PCa.

To better appreciate the biological significance of UCA1 for its contribution to prostate carcinogenesis, we modulated expression level of UCA1 and CXCR4 in PCa cells. Our functional assays clearly showed that UCA1 and CXCR4 exerts oncogenic effect, such as promotion of proliferation and cell invasion. To decipher the ceRNA mechanism between UCA1, miR-204 and its downstream targets CXCR4. We first construct several luciferase report plasmids that contains miR-204 binding sites in the 3′-UTR region of UCA1 and CXCR4. In consistent to our hypothesis, miR-204 is able to regulate luciferase activity that containing the binding sites in UCA1 and CXCR4. Most importantly, we found overexpression of UCA1 in PCa cells completely abolished the inhibitory effect of miR-204 on CXCR4, supporting that UCA1 enhances CXCR4 through inhibition of miR-204 activity. Therefore, dual targetting UCA1 and miR-204 could provide a new therapeutic strategy to suppress this oncogenic pathway for PCa patients.

In summary, this is the first study to systematically interrogate the functional and clinical significance of UCA1 in prostate cancer, and we provide comprehensive evidence that it acts as a novel oncogenic lncRNA in PCa. From a functional perspective, UCA1 impairs tumor suppressive effects of miR-204 in PCa cells. Mechanistically, overexpression of UCA1 enhanced CXCR4 through inhibition of miR-204 activity. Therapeutic targetting of miR-204 maybe a potential treatment option for patients with PCa.

## Abbreviations

BPH: benign prostatic hyperplasia
CeRNA: competing endogenous RNA
EMT: epithelial-mesenchymal transition
IgG: immunoglobulin G
NC: negative control
PCa: prostate cancer
qRT-PCR: quantitative real time-PCR
RIP: RNA immunoprecipitation
UCA1: urothelial carcinoma associated 1
